# Machine learning applications in prostate cancer magnetic resonance imaging

**DOI:** 10.1186/s41747-019-0109-2

**Published:** 2019-08-07

**Authors:** Renato Cuocolo, Maria Brunella Cipullo, Arnaldo Stanzione, Lorenzo Ugga, Valeria Romeo, Leonardo Radice, Arturo Brunetti, Massimo Imbriaco

**Affiliations:** Department of Advanced Biomedical Sciences, University of Naples “Federico II”, Via S. Pansini, 5, 80131 Naples, Italy

**Keywords:** Machine learning, Magnetic resonance imaging, Prostate, Prostatic neoplasms, Radiomics

## Abstract

With this review, we aimed to provide a synopsis of recently proposed applications of machine learning (ML) in radiology focusing on prostate magnetic resonance imaging (MRI). After defining the difference between ML and classical rule-based algorithms and the distinction among supervised, unsupervised and reinforcement learning, we explain the characteristic of deep learning (DL), a particular new type of ML, including its structure mimicking human neural networks and its ‘black box’ nature. Differences in the pipeline for applying ML and DL to prostate MRI are highlighted. The following potential clinical applications in different settings are outlined, many of them based only on MRI-unenhanced sequences: gland segmentation; assessment of lesion aggressiveness to distinguish between clinically significant and indolent cancers, allowing for active surveillance; cancer detection/diagnosis and localisation (transition *versus* peripheral zone, use of prostate imaging reporting and data system (PI-RADS) version 2), reading reproducibility, differentiation of cancers from prostatitis benign hyperplasia; local staging and pre-treatment assessment (detection of extraprostatic disease extension, planning of radiation therapy); and prediction of biochemical recurrence. Results are promising, but clinical applicability still requires more robust validation across scanner vendors, field strengths and institutions.

## Key points

- Machine/deep learning is a powerful tool to analyse large amounts of data, also applied to prostate magnetic resonance imaging (MRI).
- Differences in the pipelines for applying machine and deep learning to prostate MRI exist.
- Applications of machine/deep learning to prostate MRI regarding gland segmentation, cancer detection and localisation, assessment of lesion aggressiveness, local staging and pre-treatment assessment, and prediction of biochemical recurrence.
- Many of these applications are based only on MRI-unenhanced sequences.

## Background

Prostate cancer (PCa) represents the most common cancer in the male population, and its early detection is fundamental to reduce mortality [1]. Since only a part of PCa cases are clinically significant (csPCa), risk stratification is of crucial importance in order to avoid overdiagnosis and overtreatment. While for biopsy-naïve patients this has been performed employing clinical and laboratory parameters, imaging, especially magnetic resonance imaging (MRI), has acquired an increasingly important role in this task [2].

**Fig. 1:**
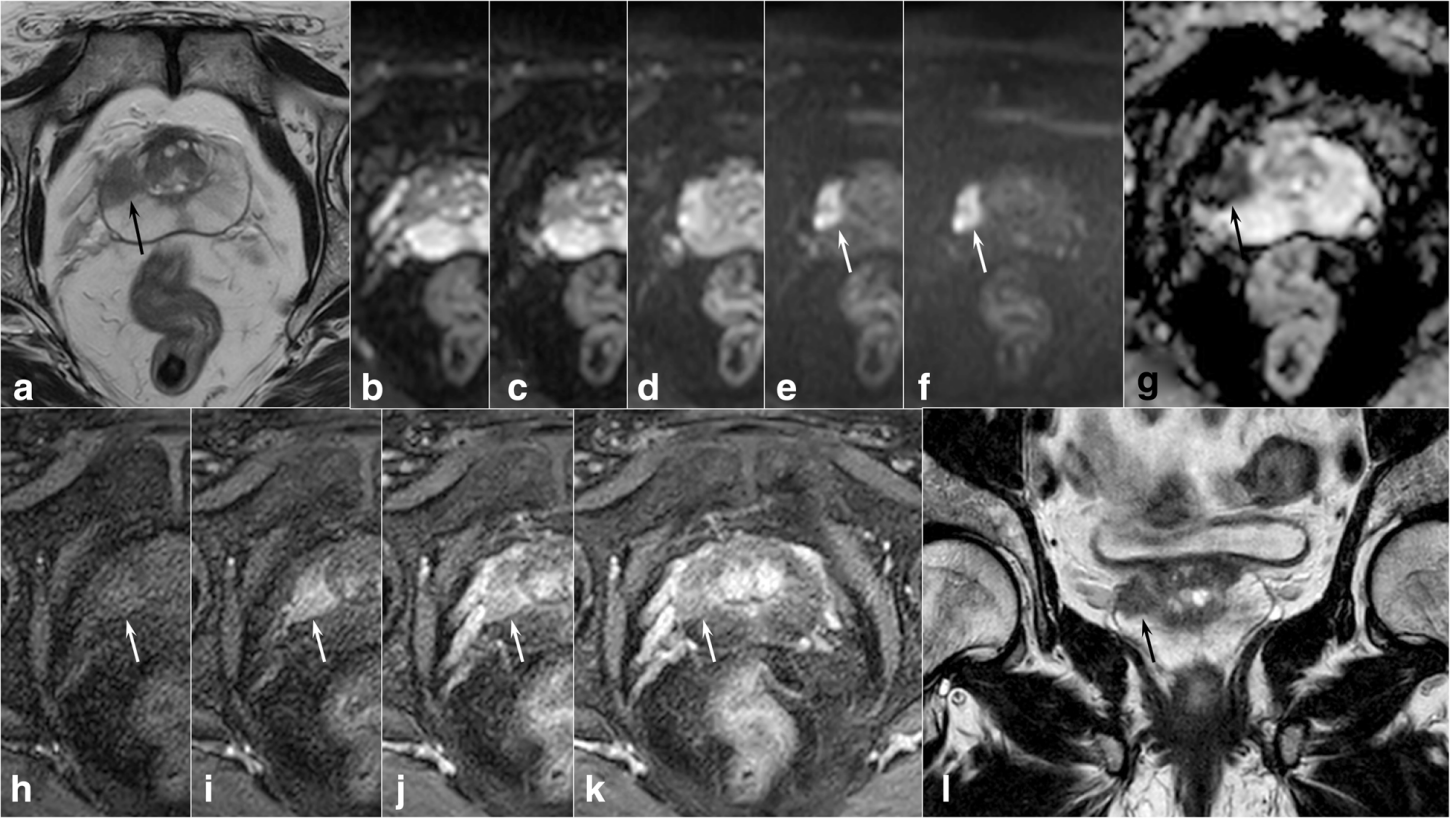
Prostate multiparametric magnetic resonance imaging showing a neoplastic lesion of the right peripheral zone (arrows). The lesion is hypointense on axial (**a**) and coronal (**l**) T2-weighted images and demonstrates diffusion restriction on diffusion-weighted images (*b* values 0, 100, 500, 1000, and 1400 s/mm^2^, from **b** to **f**, respectively), confirmed by the apparent diffusion coefficient map (**g**). Lesion enhancement is also evident on dynamic contrast-enhanced perfusion-weighted imaging (from **h** to **k**). PI-RADSv2 diagnostic category: 5

In the past, transrectal ultrasound was the main imaging technique for assessment of patients with suspected PCa, but it presents numerous limitations, with both low sensitivity and specificity rates, especially for transition zone lesions [2]. More recently, multiparametric MRI (mpMRI) has demonstrated a better diagnostic accuracy and is becoming a clinical routine examination for patients at risk of having csPCa [3–5]. The second version of the Prostate Imaging Reporting and Data System (PI-RADS) was recently updated both in regard to minimum technical acquisition parameters and image interpretation [6]. It describes a standard prostate mpMRI protocol that combines anatomical T2-weighted images with one or more functional sequences, *i.e.*, diffusion-weighted imaging (DWI) and/or dynamic contrast-enhanced (DCE) sequences (Fig. 1). In short, DWI, together with apparent diffusion coefficient (ADC) maps, is the dominant sequence to detect and establish the aggressiveness of peripheral zone (PZ) lesions. On the other hand, T2-weighted images are the most useful tool for diagnosing tumours of the transition zone. DCE has a relatively minor role as it is mainly used for the characterisation of PZ lesions in conjunction with DWI and ADC maps [7–9]. It has also been found that the use of mpMRI-targeted biopsies increases the accuracy of diagnosing csPCa and reduces the number of patients requiring repeat biopsies when compared to transrectal ultrasound-guided biopsies [4, 10].

Nonetheless, mpMRI still presents some limitations. In particular, variability is reported in terms of inter-reader agreement and diagnostic accuracy, mainly dependent on reader experience [11–14].

Machine learning (ML) is a branch of data science, and in particular of artificial intelligence, based on the development and training of algorithms, by which computers may learn from data and perform predictions without previous specific programming. The main difference with classical rule-based algorithms is represented by their ability to take advantage of increased exposure to large and new data as well as to improve and learn over time. We can identify three different types of ML algorithms [15]:

1. *Supervised learning*, the most used in radiology, which depends on train data labelling prior to the learning process
2. *Unsupervised learning*, characterised by the absence of preliminary human division of data in categories
3. *Reinforcement learning*, in which the algorithm learns from both its mistakes and successes, thanks to a continuous feedback

The main strength of ML is its ability to analyse and employ an enormous quantity of data, much more efficiently than possible for humans through classical statistical analyses. Therefore, it is not surprising that its increased role in radiology has followed the growing role and potential shown in research by radiomics. This is another expanding field that allows the extraction of great volumes of quantitative data from medical images [16]. These large datasets have been analysed to obtain useful clinical information such as correlation to other biomarkers, patient prognosis or treatment outcome [17–19].

A particular application in the field of ML is that of *deep learning*. This method exploits algorithms, also called networks, that are structured in order to somewhat mimic human neural structure [20]. Briefly, for its application in the field of medical imaging analysis, data is transformed in feature vectors, derived from its voxels, which then constitute the input neurons of the network. Between the input and output strata of the algorithm, a variable number of hidden layers, also made up by neural nodes, with various structures can be implemented. Each node, represented by a numerical value, is connected to those in other layers with different strengths (or weights), leading to the output neurons that encode the final outcome [21].

In recent years, ML has been proposed for a wide range of applications in medical imaging. The most common are the detection and characterisation of neoplastic lesions in different anatomical regions [22–25]. On the other hand, it also has many other possible uses including for example acquisition time reduction, organ and lesion automated segmentation and early detection of neurodegenerative disorders [26–30]. Unfortunately, ML algorithms are still far from a widespread application in clinical practice, mainly due to the current unavailability of the large quantity of data that would be necessary for their validation. Some solutions to this issue have already been proposed. For example, Pinto dos Santos et al. [31] focused on the essential need to create structured reports which could greatly improve the quality and the reproducibility of data available to refine artificial intelligence algorithms.

There is a broad interest in the applications of ML to prostate imaging. The purpose of this article is to review the various approaches proposed in the recent literature for gland segmentation, PCa detection, lesion aggressiveness assessment, local staging, pre-treatment assessment and follow-up.

## Machine learning pipelines for prostate MRI

A typical ML post-processing pipeline applied to prostate MRI for radiomic analysis may be constituted by:

1. mpMRI examination: T2-weighted sequences, diffusion-weighted imaging (DWI) with apparent diffusion coefficient (ADC) maps and dynamic contrast-enhanced (DCE) sequences
2. Image segmentation through the delineation of regions of interest (ROIs), which can include whole gland volume, a specific zone or one or multiple lesions
3. Image pre-processing: voxel grey value normalisation (when using non-quantitative images, *i.e.*, T2-weighted, DWI, or DCE sequences), decomposition filtering for the creation of additional mineable data (*e.g.*, Laplacian of Gaussian)
4. Feature extraction from the ROI: shape, histogram, and texture (second-order features) parameters
5. Integration of radiomic data with clinical, laboratory, prognostic, and/or genomic data
6. Feature selection in relation to the class of interest
7. Algorithm training and testing
8. Validation on an external population

Alternatively, a deep learning approach would only require:

1. mpMRI examination: T2-weighted sequences, DWI with ADC maps, and DCE sequences
2. Annotation of the ROI or of the whole image, according to the desired classification output
3. Algorithm training and testing
4. Validation on an external population

**Fig. 2:**
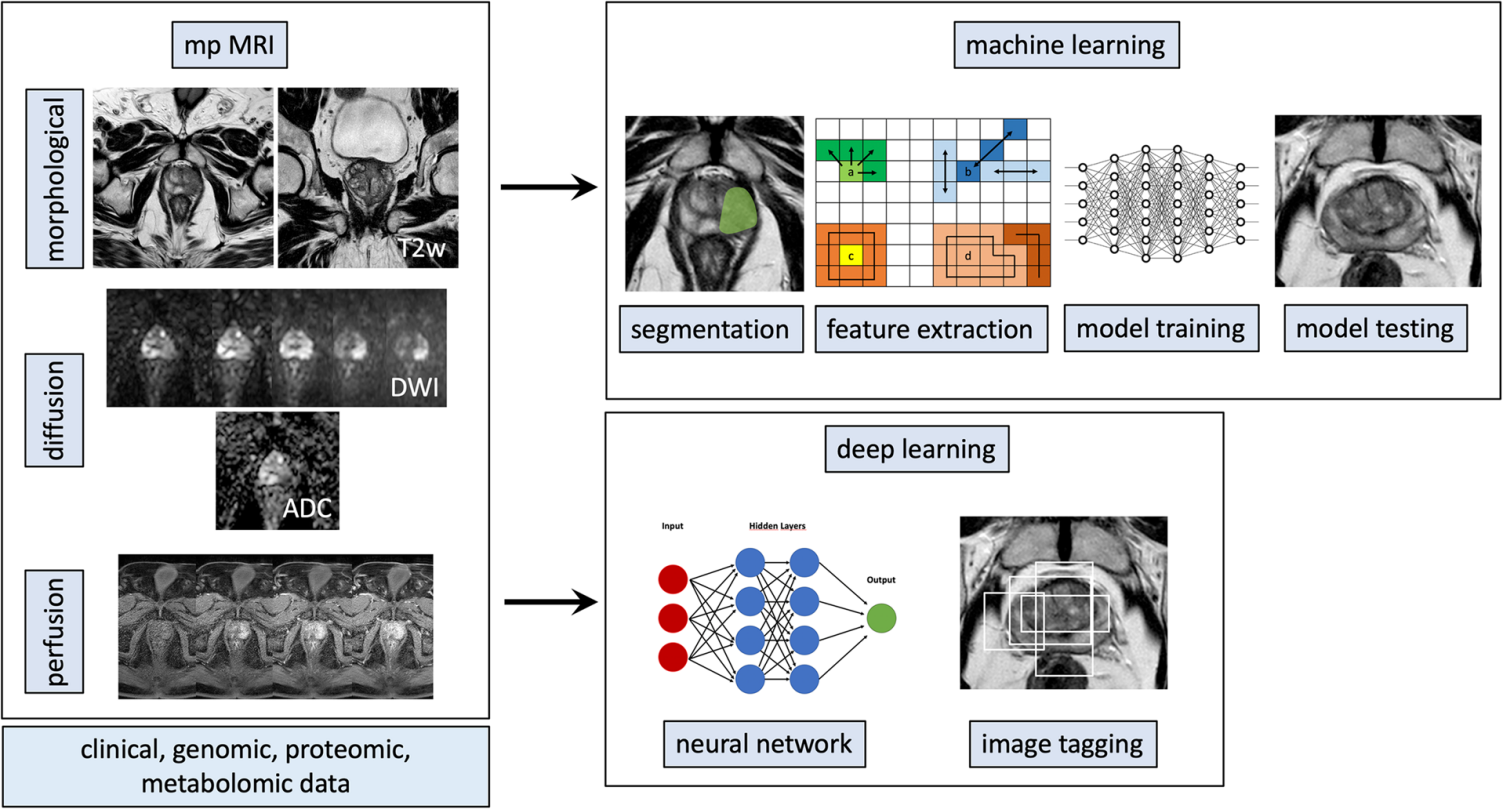
Radiomic workflow pipeline for both machine learning and deep learning approaches for prostate magnetic resonance imaging. See the text for details

An overview of both approaches is shown in Fig. 2. While the deep learning approach may appear simpler and more flexible, it does require much larger quantities of data for algorithm training and its structure is usually more complex and less transparent. These latter characteristics contribute to the ‘black box’ nature of deep learning algorithms, one of the main limitations preventing their widespread adoption. Finally, the two approaches can be variously mixed and matched combining for example radiomic data with deep learning algorithms.

## Applications

### Segmentation

The segmentation of the prostate gland is often a necessary step in clinical settings as well as for further image analysis. Therefore, the possibility of automating whole prostate as well as lesion segmentation is of great interest for the potential time-saving and increased reproducibility. A robust automated segmentation could in turn lead to a fully automated post-processing pipeline.

There are interesting studies in the literature that describe the potentials of deep learning to achieve this goal. For example, Wang et al. [32] compared manual segmentation to a novel deep learning-based one. They were able to demonstrate the feasibility of three-dimensional fully convolutional networks and subsequently validate their findings on a public dataset. Another research group [33] proposed a model based on a propagation deep neuronal network by which data from different levels of complexity were extracted and combined so as to obtain a more trustworthy segmentation of the gland and its boundaries. The propagation deep neuronal network was able to outperform baseline deep neural networks, resulting competitive with the reference standard.

Finally, Alkadi et al. [34] applied a unimodal deep learning-based system using only T2-weighted images for the automated segmentation of both the prostate and PCa lesions. Their algorithm was able to achieve an area under the receiver operating characteristic curve (AUC) of 0.995, comparable to that of other multimodal systems in lesion detection while outperforming other proposed two-dimensional and three-dimensional prostate segmentation approaches.

### Cancer detection

There is a high interest in analysing the usefulness of ML-based computer-assisted diagnosis (CAD) software in the field of PCa, as it could improve radiologists’ diagnostic performances and reproducibility. For example, already in 2012, PZ maps created by a decision support system model based on endorectal mpMRI appeared to be a promising tool in correctly localising PZ tumours [35].

More recently, Kwak et al. [36] have demonstrated that it is possible to employ radiomics and ML for the analysis of different tissues and cellular densities in the prostate gland to aid in PCa detection. In order to do so, they developed an MRI-based patient-specific prostate mould in order to ensure correspondence of prostatectomy specimens to the images. A significant difference was found in ML-determined prostatic tissue composition between benign and malignant areas. In 2018, McGarry et al. [37] found that the data derived from ten patients was sufficient to obtain a stable fit for ML MRI detection of increased epithelium and decreased lumen density areas, indicative of high-grade PCa. These authors were therefore able to generate lesion detection maps, also validated against whole-mount histopathological specimens oriented with three-dimensional printed slicing moulds.

Another proposal has been based on volumetric ROI analysis of index lesions on mpMRI [38]. These authors evaluated the usefulness of histogram parameters obtained from T2-weighted, DWI and DCE images in combination with a support vector machine (SVM) ML approach for the improvement of PI-RADSv2 scores, significantly increasing the radiologist’s performance. In a recent study [39], comparing the analysis of ADC radiomics with ML analysis and the performance of mean ADC values alone for differential diagnosis of benign and malignant prostate lesions, the resulting accuracy was similar without a statistically significant difference between approaches.

Ginsburg et al. [40] have suggested that different ML predictive models should be developed for the transition zone and PZ, as lesions and normal prostatic tissue have different imaging characteristics in these zones. In particular, they compared two zone-specific algorithms, trained on different radiomic feature datasets, with a zone-ignorant one. Interestingly, they found that while a significant difference in performance was found between the PZ-specific algorithm and the zone-ignorant one, no differences were found for the transition zone-specific algorithm.

A further use case for ML is the differentiation of stromal benign prostatic hyperplasia from PCa in the transition zone. This diagnosis can be quite challenging, especially with small lesions. Statistical analysis of previously established quantitative features (ADC maps, shape, and image texture) demonstrated a high accuracy in the differentiation of small neoplastic lesions from benign ones using either linear regression and SVM classifiers [41].

Another potential advantage of ML CAD-assisted mpMRI is the improvement of inter-reader agreement. Greer et al. [42] compared index lesion sensitivity, specificity and agreement between eight radiologists from different institutions and with various levels of experience. The readers, after a first qualitative examination, reanalysed the same images using CAD: agreement and sensitivity were higher in the CAD-assisted mpMRI, but specificity worsened. Furthermore, the system was more useful for PZ lesions.

Another CAD system was developed by Ishioka et al. [43], although their proposal was based on a convolutional neural network deep learning algorithm. While the accuracy reported in two validation sets was not very high, with AUC respectively of 0.645 and 0.636, further development could lead to a reproducible, automated CAD system. On the other hand, the research unit lead by Wang [44] has shown better results for a deep learning-based fully automated segmentation when compared to a non-deep learning model. The authors assessed their ability to distinguish PCa from benign pathologies such as prostatitis or prostate benign hyperplasia. Their results showed a better accuracy and reliability of deep learning, with a statistically significantly higher AUC than that of the non-deep learning model (0.84 vs. 0.70). Interestingly, their approach was conducted without any need for segmentation of the training set, potentially easing further evolution of their software with added data.

### Assessment of lesion aggressiveness

As PCa is frequently indolent, it is of great importance to assess the aggressiveness of detected lesions, and therefore their clinical significance, for patient management [2]. The standard approach of MRI and subsequent targeted biopsy have improved identification of csPCa foci, but significant disease is still missed, as shown in a recent meta-analysis [45]. Texture features have shown in the past potential as biomarkers of PCa aggressiveness [46, 47].

An interesting study was performed on 56 PCa patients in active surveillance who underwent MRI-guided biopsies [48]. The authors assessed differences between patients who were biopsy- and MRI-negative and patients who were biopsy- and MRI-positive, in radiomic features extracted from T2-weighted and ADC images. They subsequently employed the ten selected parameters to construct models to identify subjects who were biopsy-positive while MRI-negative and those biopsy-negative and MRI-positive. Quadratic discriminant analysis enabled to obtain the best accuracy improving, compared to PI-RADS alone, by 80% for the first classification and 60% in the second. While focusing only on the central gland, Li et al. [49] have shown that an SVM approach, trained on six features extracted from mpMRI exams depicting 152 prostate lesions, was able to consistently predict Gleason score. This is especially important as it is one of the main determinants of PCa clinical significance.

For the task of distinguishing indolent PCas and csPCas, deep learning methods have shown some potential for future applications. Zhong et al. [50] compared both a deep learning and a deep transfer-learning algorithm to the performance of PI-RADSv2. In their validation cohort of 30 patients with 47 lesions, it was comparable to the radiologist’s assessment (AUC 0.73 and 0.71, respectively), while outperforming deep learning alone (AUC 0.69). Similarly, Yuan et al. [51] have shown that transfer-learning, in their case applied to the AlexNet neural network, was able to achieve an overall accuracy of 87% for the prediction of lesion Gleason score.

Finally, a novel approach for the detection of csPCa, as defined in the National Comprehensive Cancer Network guidelines, using radiomics in combination with ML, was recently performed by Varghese et al. [52]. A framework for the robust testing of seven ML algorithms with fivefold cross-validation showed that the best classifier was a quadratic kernel-based SVM with an overall accuracy of 0.92 in the validation cohort.

### Local staging and pre-treatment assessment

mpMRI also gives important information for pre-treatment local staging. In this setting, proposals for artificial intelligence applications have been more limited. A recent preliminary report has shown that a radiomics-based Bayesian network achieved a high accuracy (AUC 0.88) in the detection of extraprostatic extension of disease in preoperative MRI, using radical prostatectomy as the reference standard [53]. This finding is promising for further development of ML in PCa local staging.

Some novel applications of ML to prostate MRI have been proposed in the setting of treatment planning. In particular, Sun et al. [3] have developed a new type of focal radiotherapy (bio-focused therapy) that requires prior evaluation of some biological features of the tumour, such as cell density and aggressiveness, which relate to therapy response. These have been assessed by the authors non-invasively, with a voxel-wise approach: patients underwent mpMRI before radical prostatectomy, and imaging data were combined with histopathological information to extract tissue features (cell density). They were then able to create models of prostate tissue cell density, used for the targeting of bio-focused therapy in order to achieve better response and lower toxicity. Shafai-Erfani et al. [54] trained a ML algorithm with paired CT and MRI datasets in order to generate synthetic CT images to be used for patient radiation therapy setup and dose calculation. ML proved capable of producing reliable CT images, comparable to the ground truth for both tasks.

### Biochemical recurrence

About 30% of PCa relapse after radical prostatectomy [55]. Also, radiation therapy-resistant PCa or recurrent PCa is not uncommon. The elevation of serum levels of prostate-specific antigen after treatment is currently the most employed biomarker of this condition and is known as ‘biochemical recurrence’ [56]. Very complex altered molecular networks lie behind it and, more extensively, PCa recurrence [55]. ML applied to mpMRI has been proposed as a viable tool for early detection of recurrence or prediction of treatment outcome.

Abdollahi et al. [57] investigated how mpMRI ML models could predict the response to an intensity-modulated radiation therapy. Their findings suggest that both pre- and post-treatment radiomic features can give a reliable prediction of therapeutic success, especially if compared to prostate-specific antigen dosage. A recent study [58] has shown that a SVM could accurately predict biochemical recurrence within 3 years of radical prostatectomy, outperforming a linear regression model based on both MRI and the D’Amico patient risk classification. In fact, ML showed an overall accuracy of 92.2% compared to the 79% achieved by linear regression. The potential of ML for this task was confirmed in 2018 by another study [59] that employed SVM, linear discriminant analysis and random forest on radiomic features extracted from T2-weighted and ADC images.

## Conclusions

A number of studies showed that ML, with or without radiomic feature extraction, has a great potential to improve the diagnostic performance and to expand the clinical role of prostate MRI. Its applications range from segmentation, lesion detection and aggressiveness prediction to local staging and assessment prior to and following treatment. It is interesting to note that many of the cited studies do not employ DCE images. Thus, ML could help in avoiding the systematic use of contrast agents for prostate imaging, as suggested by current guidelines, a development to monitor as gadolinium administration has come under scrutiny for the growing evidences of its accumulation in the body [60].

Clinical applicability still requires more robust validation across scanner vendors, field strengths and institutions, as for ML in all fields of medical imaging. As highlighted in a recent letter by Peter L. Choyke, the current limitations of ML also prevent them from gaining the trust of the ideal end-users, radiologists [61]. On the other hand, the growth in quantity and quality of research in the years is also undeniable, and as ML software obtain approval for clinical use in other settings, it is difficult to imagine a future for prostate MRI without it.

## Data Availability

Not applicable.

## Abbreviations

ADC: Apparent diffusion coefficient
AUC: Area under the curve
CAD: Computer-assisted diagnosis
csPCa: Clinically significant prostate cancer
DCE: Dynamic contrast-enhanced
DWI: Diffusion-weighted imaging
ML: Machine learning
mpMRI: Multiparametric MRI
MRI: Magnetic resonance imaging
PCa: Prostate cancer
PI-RADS: Prostate Imaging Reporting and Data System
PZ: Peripheral zone
ROI: Region of interest
SVM: Support vector machine
